# The Effects of a Novel Non-catechol Dopamine Partial Agonist on Working Memory in the Aged Rhesus Monkey

**DOI:** 10.3389/fnagi.2021.757850

**Published:** 2021-11-26

**Authors:** Tara L. Moore, Damon A. Young, Ronald J. Killiany, Kari R. Fonseca, Dmitri Volfson, David L. Gray, Rita Balice-Gordon, Rouba Kozak

**Affiliations:** ^1^Department of Anatomy & Neurobiology, Boston University School of Medicine, Boston, MA, United States; ^2^Center for Systems Neuroscience, Boston University, Boston, MA, United States; ^3^Internal Medicine Research Unit Pfizer Worldwide Research, Development and Medical Pfizer Inc., Cambridge, MA, United States; ^4^Medicine Design, Pfizer Worldwide Research, Development and Medical Pfizer Inc., Cambridge, MA, United States

**Keywords:** dopamine, aging, prefrontal cortex, rhesus monkey, memory

## Abstract

Aged-related declines in cognition, especially working memory and executive function, begin in middle-age and these abilities are known to be mediated by the prefrontal cortex (PFC) and more specifically the dopamine (DA) system within the PFC. In both humans and monkeys, there is significant evidence that the PFC is the first cortical region to change with age and the PFC appears to be particularly vulnerable to age-related loss of dopamine (DA). Therefore, the DA system is a strong candidate for therapeutic intervention to slow or reverse age related declines in cognition. In the present study, we administered a novel selective, potent, non-catechol DA D1 R agonist PF-6294 (Pfizer, Inc.) to aged female rhesus monkeys and assessed their performance on two benchmark tasks of working memory – the Delayed Non-match to Sample Task (DNMS) and Delayed Recognition Span Task (DRST). The DNMS task was administered first with the standard 10 s delay and then with 5 min delays, with and without distractors. The DRST was administered each day with four trials with unique sequences and one trial of a repeated sequence to assess evidence learning and retention. Overall, there was no significant effect of drug on performance on any aspect of the DNMS task. In contrast, we demonstrated that a middle range dose of PF-6294 significantly increased memory span on the DRST on the first and last days of testing and by the last day of testing the increased memory span was driven by the performance on the repeated trials.

## Introduction

Age-related decline in working memory and executive function have been well documented in both humans and monkeys (Moore et al., 2003, 2006; Alexander et al., 2012; Bizon et al., 2012; Murman, 2015; Salthouse, 2016). Working memory and executive function are mediated by the prefrontal cortex (PFC) and more specifically through the normal function of the dopamine (DA) system within the PFC (Mishkin and Pribram, 1956; Mishkin, 1957; Rosvold et al., 1961; Sawaguchi and Goldman-Rakic, 1991; De Bundel et al., 2013; Takahashi, 2013; Wass et al., 2013; Arnsten et al., 2015; Burke et al., 2018; Wang et al., 2019; Soutschek et al., 2020a,b). There is a significant decrease in DA levels with age (Sawaguchi et al., 1990; Sawaguchi and Goldman-Rakic, 1991; Arnsten et al., 1994; Thierry et al., 1998; Volkow et al., 1998, 2000) and the PFC appears to be particularly vulnerable to age-related loss of dopamine with a 56% decrease in DA neurotransmitter concentration in monkeys 18 years and older (Goldman-Rakic and Brown, 1981; Arnsten et al., 1994). Numerous studies have also demonstrated age-related changes in DA receptors (Brozoski et al., 1979; Arnsten et al., 1994, 1995; Volkow et al., 1996, 1998, 2000; Cai and Arnsten, 1997; Wang et al., 1998; Moore et al., 2005; Bäckman et al., 2006, 2010; Morcom et al., 2010). Evidence from PET scans have shown an age-related decline in DA receptor densities in the striatum and frontal cortex of the aging brain (Bäckman et al., 2011; Karlsson et al., 2011; Rieckmann et al., 2011) and concentrations of D1 receptors are related to performance on tasks of working memory and executive function in middle-age and aged humans and monkeys (Arnsten et al., 1994, 1995; Cai and Arnsten, 1997; Müller et al., 1998; Castner and Goldman-Rakic, 2004; Moore et al., 2005; Bäckman et al., 2011; Rieckmann et al., 2011). Further, it has been demonstrated that administration of DA agonists to aged monkeys improves working memory (Cai and Arnsten, 1997; Castner and Goldman-Rakic, 2004) while administration of DA antagonists decreases performance on working memory tasks (Fischer et al., 2010).

Based on the studies described above, it seems likely that age associated deficits in cognition may be related to loss of DA signaling and therefore this system is a strong candidate for therapeutic intervention to slow or reverse these declines. For example, the administration of a DA D1 receptor antagonist, ecopipam, altered performance in aged rodents on choice tasks and their performance was partially enhanced by co-administration of D1 agonists (Yohn et al., 2015). Recently, Wang et al. (2019), demonstrated that the DA D1 agonist PF-3628, which has a moderate affinity for the DA D1 receptor, when applied directly to DLPFC neurons in aged monkeys performing a delay dependent working memory task caused enhanced task related firing. Building on these promising findings, in the present study, we administered a novel selective, potent, non-catechol DA D1 R partial agonist PF-6294 (Pfizer, Inc.) to aged female rhesus monkeys and assessed their performance on two benchmark tasks of working memory. PF-6294 is structurally very similar to other D1 partial agonists recently described by Pfizer (Gray et al., 2018; Wang et al., 2019; Kozak et al., 2020; Young et al., 2020). This compound was chosen based on its suitable non-human primate pharmacokinetics and its D1 pharmacology, including affinity for human D1 receptors (Ki = 17 nM), functional partial agonism at human D1 receptors (EC50 = 36 nM, Intrinsic activity = 69% relative to dopamine), and in particular, binding to the non-human primate D1 receptor (Ki = 14 nM). We designed the experiment to assess both acute and repeat dose effects on two different tasks to report on subcomponents of working memory performance. We also tested a range of doses with an overall goal of building on our understanding of the role of D1 receptor activation in specific aspects of the working memory process.

## Materials and Methods

### Subjects

Eight, behaviorally naive, middle aged-old (17–23 yrs of age; equivalent to approximately 51–69 human years), female rhesus monkeys (*M. mulatta*) were used in the present study. All of the monkeys were obtained from national primate research facilities or private vendors and had known birth dates and complete health records. Before entering the study, monkeys received complete medical examinations. In addition, all monkeys underwent magnetic resonance imaging (MRI) to ensure there was no occult neurological damage prior to the start of the study. Results of the medical exams and MRIs revealed that all monkeys were healthy at the time of the study. While on study, monkeys were individually housed in colony rooms where they were in constant auditory and visual range of other monkeys in the Animal Science Center (ASC) of Boston University School of Medicine. This facility is fully AAALAC accredited and all research protocols were approved by the Boston University Institutional Animal Care and Use Committee.

Diet consisted of Purina Monkey Chow (Purina Mills Inc, St. Louis, MO, United States) supplemented by fruit, vegetables and forage with feeding taking place once per day, immediately following behavioral testing. Water was available continuously. The monkeys were housed under a 12-h light/dark cycle with cycle changes occurring in a graded fashion over the course of an hour. Following a mandatory 6-week quarantine period and acclimation to the colony room, animals were ready to begin pre-training on cognitive tasks.

### Binding to D1 in Non-human Primates

PF-6294 and other test compounds were synthesized as free bases by Pfizer worldwide medicinal chemistry. For PF-6294, batch number 0001 was tested. All compounds were dissolved in 100 percent DSMO and stored in a Nitrogen environment. Human D1 binding affinity and Human cAMP functional activity were determined according to a previously described method (Gray et al., 2018). D1 binding studies in non-human primate striatal tissue were conducted as follows.

Compounds were titrated to assess binding as a 10 point concentration response. Radioligand was purchased from Perkin Elmer (Boston, MA, United States). Non-specific binding controls were purchased commercially. Primary striatal tissue from a cynomolgus monkey brain was used for receptor binding studies. The radioligand was [3H]-SCH23390 (1.0 nM) and the tissue concentration was 9 mg/mL. The Kd of the radioligands used for these binding experiments was 1.7 nM, determined in separate saturation experiments.

Tissue preparation buffer was 50 mM Tris buffer containing 2.0 mM MgCl2 at pH 7.4 (at 4°C). Monkey striatal tissue was homogenized in tissue buffer using a Polytron (setting 5) and centrifuged at 40,000*g* for 10 min. Tissue was re-suspended in tissue preparation buffer using a polytron and centrifuged for 10 min at 40,000*g*. The resulting pellet was then re-suspended in 50 mM Tris HCl with 4 mM MgSO4 and 0.5 mM EDTA assay buffer at pH 7.4 at room temperature.

Test compounds (including PF-6294), (+)-Butaclamol as a non-specific control, and DMSO controls (2.5 μL) were added to 96-well plates (Costar 3363) at 100-fold their final concentrations, followed by 50 μL radioligand at 5-fold the final concentration. The exact concentration of radioligand for each experiment was determined by liquid scintillation counting. Competition binding was initiated by the addition of 200 μL of tissue homogenate and incubated for 30 min at 37°C. After incubation, assay samples were rapidly filtered through Unifilter-96 GF/B PEI-coated filter plates (Perkin Elmer, Boston, MA, United States) using a plate harvester (Packard) and rinsed with ice-cold 50 mM Tris buffer (pH 7.4 at 4°C). Filter plates were dried overnight at room temperature. Plates were bottom sealed prior to the addition of 50 μL/well Ecolume (MP Biomedicals, Solon, OH, United States) scintillation fluid. Plates were then top sealed and read on a Trilux (Perkin Elmer, Boston, MA, United States). Percent effects for each concentration were determined, and IC50 values were calculated using a logistic 4 parameter fit model. Ki values were calculated from the IC50 values using the Cheng-Prusoff equation: Ki = IC50/(1 + ([L]/Kd)), where L is the concentration of the radioligand used in the experiment; Kd is the affinity of the radioligand (determined in separate saturation experiments). Additional proprietary and reference compounds were also included in each run to confirm assay consistency.

### Drug/Vehicle Administration

Three doses of drug (0.002, 0.004, and 0.012 mg/kg) were used in the present study. The doses for the current study were selected based on initial subcutaneous pharmacokinetic study data showing linear PK across a similar dose range following SC administration (data is not reported). At the chosen doses the exposure was predicted to cover a wide range of D1 receptor occupancy.

Drug was dissolved in 5% dimethyl sulfoxide + 5% Cremophor EL + 90% sterile water or sterile saline + 0 to 3 molar equivalents of hydrochloric acid to a pH ∼3–4 for subcutaneous administration. Total administration volume ranged from 2 to 10 ml depending on the weight of the individual monkey.

The study was divided into three drug/testing sessions and each monkey received daily administration of either one of the three doses (labeled A, B or C) or the vehicle (labeled D) only during each of the three sessions. A balanced cross-over design was used and each monkey received two of the three dose levels of PF-6294 and 1 dose of vehicle across the three testing periods (see Table 1). The doses administered were randomly assigned to each monkey. Drug or vehicle were administered by subcutaneous injection in the thigh and injection sites were alternated between the left and right thighs and distributed across each thigh to prevent irritation. Drug was administrated once each day, one hour prior to beginning the daily testing session. Each drug/testing session spanned three weeks and within each session, monkeys were dosed every day for 22 consecutive days and were tested five days a week (Monday–Friday). A six-week wash-out period occurred between each drug/testing session during which no drug was administered and monkeys were not tested. All personnel were blind to treatment groups.

**TABLE 1:** Balanced cross-over design for drug administration across testing periods.

Cohort	Testing/drug administration #1	Testing/drug administration #2	Testing/drug administration #3
1 (*n* = 3)	D	B	C
2 (*n* = 2)	A	D	C
3 (*n* = 3)	A	B	D

Baseline blood samples were collected prior to entering the study and then additional blood samples were drawn at two time points during each drug/testing session. First, blood was drawn the day after the completion of a testing session. For this blood draw, the drug or vehicle was administered the morning following the last day of testing in a session (Day 22 of dosing) and then blood was drawn one hour later. Second, blood was drawn on the last day of each wash-out period. Blood samples were collected into vacutainers containing K3-EDTA and maintained on ice prior to centrifugation. Following centrifugation, the resultant plasma samples were transferred to polypropylene tubes and stored frozen at –80°C until analysis of compound concentrations.

### Plasma Protein Binding

The unbound fraction of PF-6294 in monkey plasma (fu,p) was determined by equilibrium dialysis using standard procedures (Di et al., 2017).

### Calculation of Receptor Occupancy

The unbound plasma concentrations (Cp,u) of PF-6294 were calculated by multiplying the total plasma concentrations (C_p_) by the fraction unbound in plasma (*f*_*u,p*_). The unbound concentrations in the plasma were presumed to be in equilibrium with the unbound brain concentrations. The receptor occupancy (RO) of PF-6294 was calculated based upon the C_p,u_ and the monkey *in vitro* K_*i*_ (14 nM) determined through use of a radioligand binding assay in monkey striatal tissue through use of the following equation:


$$\text{RO}=\frac{C_{p,u}}{\left(C_{p,u}+K_{i}\right)}\times 100$$

### Quantitation of Compound in Biological Samples

A non-validated liquid chromatography tandem mass-spectrometry (LC-MS/MS) method was used for the quantitation of PF-6294 in monkey plasma and plasma protein binding samples. All samples were subjected to protein precipitation followed by LC-MS/MS analysis to determine PF-6294 concentrations.

### Cognitive Testing

All monkeys were initially familiarized with cognitive testing in a Wisconsin General Testing Apparatus (WGTA). All monkeys were trained only to displace a single gray plaque placed pseudo-randomly over one of three lateral food wells in a wooden testing board to obtain a reward. Cut grapes, raisins and/or small pieces of candy were used as rewards during testing. Monkeys were trained until they responded for 40 consecutive trials on two successive days.

### Baseline Testing

**FIGURE 1:**
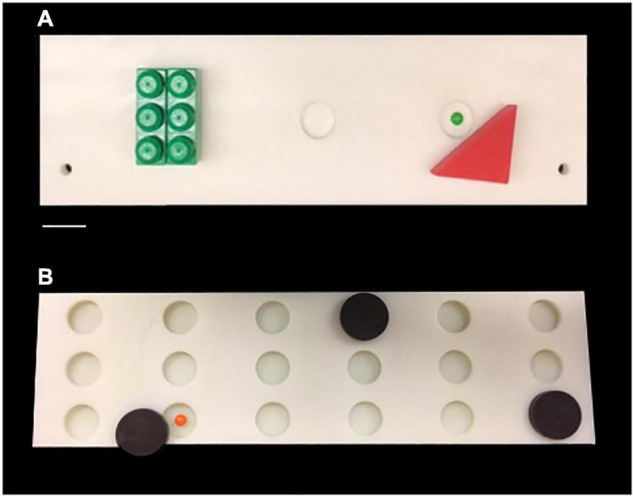
**(A)** Photograph of the testing board used for Delayed Non-match Sample Task. **(B)** Photograph of the testing board used for Delayed Recognition Span Task. Scale Bar = 5.0 cm.

Monkeys were then trained on the Delayed Non-matching to Sample task (DNMS). The DNMS task is a benchmark task of recognition memory that is widely used with non-human primates (Figure 1A). The DNMS task assesses the monkey’s ability to identify a novel from a familiar stimulus. Each trial of DNMS begins with a sample object presented over the central baited food well of the same wooden testing board used during testing familiarization. The monkey was permitted to displace the object and obtain the food reward. The door was then lowered, and the original, now familiar, sample object was placed over an unbaited lateral well and a new, novel, unfamiliar object is placed over the other lateral well that was baited. Ten seconds after the original sample trial, this choice trial was begun and the monkey must choose the unfamiliar, novel object in order to obtain the reward. Twenty seconds later, the next trial was initiated with a new, novel sample object presented over the baited central well followed 10 s later by another recognition trial using that second sample object and another new novel object. The position of the two objects on successive recognition trials was varied from left to right lateral wells in a predetermined pseudorandom order. Monkeys were trained on this task until their performance reached a criterion of 90% correct responses over 100 trials.

Following reaching criterion on the DNMS task, monkeys then completed 5 days (5 trials per day) of the **Delayed Recognition Span Task (DRST)-spatial.** The DRST-spatial task requires the monkey to identify, trial by trial, a new stimulus among an increasing array of serially presented, familiar stimuli (Figure 1B). Fifteen identical plain brown discs (6 cm in diameter) were used as stimuli. For the first sequence, one disc was placed over one of the eighteen wells, which was baited. The screen was raised and the monkey is allowed to displace the disc to obtain the reward. The screen was lowered and a second disc is placed on the board over a baited well while the first disc was returned to its original position over the now unbaited well. After 10 s, the screen was raised and the monkey was required to identify and displace the new disc that was in a novel spatial location in order to obtain the reward. Each successive correct response trial was followed by the addition of a new disc on the next trial until the monkey made an error (i.e., chooses a previously chosen disc) or reaches a span of 9 correct responses. With the occurrence of the first error, the trial was terminated and the number of discs on the test tray minus one constitutes the recognition span score for that trial (i.e., number of correct responses). One trial each day was a single repeated sequence that was included in the test administration based on Hebb’s Repetition Paradigm that describes that the serial recall of a repeated sequence of information or stimuli gradually develops into stable, durable and longer-term memory (Cohen and Johansson, 1967; Szmalec et al., 2009). Therefore, quantifying the span on repeated and non-repeated trials allows us to determine if there is evidence of greater learning and retention of a repeated sequence even with in the presence of an impairment in spatial working memory on the non-repeated trials. The total span was calculated by taking the mean total span across the five days. The total repeated span was calculated by taking the mean of the span achieved on the repeated sequence each day. The total non-repeated span is calculated by subtracting the repeated span from the total span.

### Testing During Drug/Test Sessions

During each drug/test session, we administered three tasks: DNMS Basic, DNMS delays (with and without interference) and Delayed Recognition Span Task (DRST).

### Delayed Non-matching-to-Sample (DNMS – Basic Acquisition)

During the 1st week of testing in each testing session, the DNMS task was administered as described above for 20 trials per day for five days. The total errors made by the monkey during the 100 trials was recorded (Table 2).

**TABLE 2:** Data for individual monkeys – Delayed Non-match to Sample.

		Initial acquisition		1st Re-test				2nd Re-test				3rd Re-test			
AM #	Age entered study	DNMS total trials	DNMS total errors	DNMS total trials	DNMS total errors	DNMS 5 min delays % correct	DNMS 5 min delays % correct – interference	DNMS total trials	DNMS total errors	DNMS 5 min delays % correct	DNMS 5 min delays % correct – interference	DNMS total trials	DNMS total errors	DNMS 5 min delays % correct	DNMS 5 min delays % correct – interference
AM320p	20.7	640	193	100	5	72	84	100	7	68	72	100	7	60	80
AM331p	22.9	327	105	100	7	80	80	100	12	56	40	100	7	60	68
AM328p	19.6	903	332	100	17	64	64	100	26	64	60	100	31	56	36
AM325p	22.7	760	183	100	16	68	84	100	15	72	72	100	10	72	68
AM332p	21.0	1,313	378	100	10	64	76	100	11	80	68	100	10	76	68
AM335p	17.4	920	311	100	19	60	60	100	25	64	60	100	24	80	60
AM323p	19.7	860	202	100	15	64	80	100	11	72	76	100	18	52	72
AM339p	18.7	596	187	100	11	72	60	100	16	60	52	100	6	52	60

### Delayed Non-matching-to-Sample Delay (DNMS – 5-min Delay)

During the 2nd week of each drug/test session, a DNMS delay task was administered. DNMS delays utilizes the same concept of the DNMS basic task, but instead of a 10-s delay between presentation of the sample and choice objects there was a 5-min delay after the sample object is initially presented and before the choice trial. Ten trials were administered with the first 5 trials administered only with 5-min delays and the last five trials also included an “interference” component. The interference component involved administering one trial of a simple color discrimination task to the monkey during each of the last five trials within the 5-min delay. Specifically, 30-s after the initial sample object was presented to the monkey, green and red plaques were placed over the left and right wells (out of sight of the monkey) and the monkey had to choose one of the plaques. For all monkeys the “red” plaque was always correct on all trials. Then at the end of the 5-min delay, the choice trial in the original DNMS delay trial was administered. The total number of correct responses during the non-interference and the interference trials were recorded for each monkey (Table 2).

### Delayed Recognition Span Task-Spatial

During the 3rd week of each drug/test session, the monkeys completed the DRST-spatial task. Five trials were presented each day for five consecutive days (25 trials) during each test session. One trial each day was a single sequence that was repeated each day (5 trials). The total span was calculated by taking the mean total span across the five days. The total repeated span was calculated by taking the mean of the span achieved on the repeated sequence each day. The total non-repeated span is calculated by subtracting the repeated span from the total span (Table 3).

**TABLE 3:** Data for individual monkeys – Delayed Recognition Span Test.

			Initial acquisition			1st Re-test			2nd Re-test			3rd Re-test		
Cohort	AM #	Age entered study	DRSTsp total span	DRSTsp non-repeat span	DRSTSP repeat span	DRSTsp total span	DRSTsp non-repeat span	DRSTsp repeat span	DRSTsp total span	DRSTsp non-repeat span	DRSTsp repeat Span	DRSTsp total Span	DRSTsp non-repeat span	DRSTsp repeat span
1	AM320p	20.7	2.52	2.3	3.4	2.08	2	2.4	2.12	2	2.6	1.84	1.75	2.2
1	AM331p	22.9	2.56	2.1	4.4	2.4	2.05	3.8	2.4	2.2	3.2	2.76	2.3	4.6
1	AM328p	19.6	1.6	1.3	2.8	1.68	1.45	2.6	1.84	1.6	2.8	1.52	1.4	2
2	AM325p	22.7	1.64	1.45	2.4	1.92	1.75	2.6	1.92	1.75	2.6	1.68	1.6	2
2	AM332p	21	1.8	1.6	2.6	2.08	1.8	3.2	1.92	1.85	2.2	2.36	2.05	3.6
3	AM335p	17.4	1.8	1.7	2.2	1.76	1.6	2.4	1.68	1.55	2.2	1.76	1.7	2
3	AM323p	19.7	1.92	1.7	2.8	2.2	1.95	3.2	2.32	1.8	4.4	2.04	1.95	2.4
3	AM339p	18.7	1.92	1.75	2.6	1.88	1.75	2.4	2.68	2.55	3.2	2	1.85	2.6

### Data Analysis

Data were analyzed using R 3.2.3 statistical software. Performance on basic and delayed with and without interference tasks were used as endpoints for DNMS. Total span, non-repeat span and repeat span were used as endpoints for the DRST. Performance on the DRST and DNMS tasks was analyzed using repeated measures mixed model ANOVA. The effects of baseline, period, treatment, day, and treatment by day interaction were included as fixed factors, while monkey and monkey by treatment interaction served as random factors. The model was fitted using the method of restricted maximum likelihood, and significance of fixed effects was evaluated using Kenward-Roger degrees of freedom estimation. Significant ANOVA results were followed by Dunnett’s post-hoc comparisons against control group, separately at each time point. Data were considered statistically significant at a level of p < 0.05.

## Results

### Plasma Protein Binding

The fraction unbound of PF-6294 in monkey plasma was 0.178.

### Plasma Concentrations of PF-6294 in Monkey Plasma

The mean plasma concentrations of PF-6294 following SC administration at 0.002, 0.004, and 0.012 mg/kg were 1.00, 3.96, and 6.50 ng/mL, respectively, at 1 h post-dose. Conversion of these concentrations to unbound molar concentrations resulted in unbound plasma concentrations of 0.48, 1.89, and 3.10 nM, respectively (MW = 373.35 g/mol).

### Receptor Occupancy at 1 h Post-dose

At 1 h post-dose, the calculated D1 receptor occupancies were 3.3, 12, and 18% at the dose levels of 0.002, 0.04, and 0.12 mg/kg, respectively. The use of unbound plasma concentrations in the calculation presumes there is no hindrance to the distribution of PF-6294 across the blood-brain barrier and that unbound brain concentrations are in equilibrium with unbound plasma concentrations.

### Delayed Non-matching to Sample – Basic Task

**FIGURE 2:**
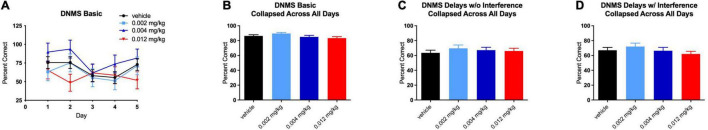
Effect of PF-6294 on performance on the basic DNMS task **(A)** on individual testing days at each dose **(B)** collapsed across all days and **(C)** on the delayed version without interference and **(D)** with interference. No dose had a significant effect on performance on any version of the task. Error bars = SEM.

For the basic version of the DNMS task, there were no significant effects on performance at any dose of PF-6294 on any individual day of testing (Figure 2A). When the data was collapsed across days, there was still no significant effect of drug at any dosing (Figure 2B). The monkeys were performing the task at >80% correct at baseline, in line with historical baseline data for similar tasks, and the presence of D1 agonist did not alter the number of correct selections. Even

### Delayed Non-matching to Sample – Delay

Similar to the lack of effect of treatment on performance in the basic DNMS task, no dose of PF-6294 had an effect on the task with increased delays (Figure 2C). The introduction of interference also yielded no significant changes in performance at any dose (Figure 2D).

### Delayed Recognition Span Task-Spatial

**FIGURE 3:**
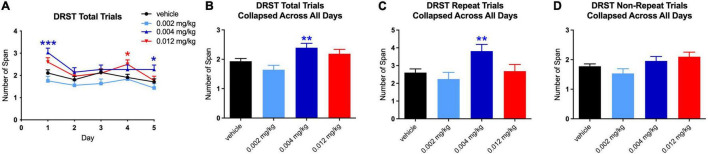
Effect of PF-6294 on DRST Spatial Overall Performance. **(A)** Analysis revealed across all days for total trials of the DRST a significantly higher span for the middle dose on day 1 (***p = 0.0002) and on day 5 (*p = 0.035). The total span was also higher on day 4 for the highest dose (red *p = 0.025). **(B)** Overall, when collapsed by day, there was a significantly higher total span achieved at the middle dose (**p = 0.003). **(C,D)** Graphs show that the increase was due to the effect on repeated trials (*p* = 0.004), but not non-repeated trials. Error bars = SEM.

For the total trials of the DRST, there were significant main effects on performance from baseline [*F*_(1,5.992)_ = 6.1216, *p* = 0.048] and treatment [*F*_(3,12.552)_ = 4.3605, *p* = 0.026], as well as an interaction between treatment and day [*F*_(16,80)_ = 3.1608, *p* = 0.0003]. *Post hoc* analysis revealed a significantly higher span, as compared to baseline, for the middle dose on day 1 [*t*_(118.51)_ = 4.164, *p* = 0.0002], as well as on day 5 [*t*_(118.51)_ = 2.531, *p* = 0.035] (Figure 3A). The total span was also higher on day 4 for the highest dose [*t*_(118.21)_ = 2.659, *p* = 0.025; see Figure 3A]. Overall, when collapsed by day, a significantly higher total span was achieved at the middle dose (0.004 mg/kg) [*t*_(117.01)_ = 3.379, *p* = 0.003; see Figure 3B]. This increase was due to the effect on repeated trials [*t*_(96.36)_ = 3.297, *p* = 0.004], but not non-repeated trials (see Figures 3C,D). While there was not a significant difference between repeated and non-repeated spans, performance on the repeated span by treated monkeys did improve across the testing sessions suggesting that although the trial was only repeated 4 times after the initial presentation, there is evidence of enhanced learning of the stimuli over repeated trials.

For repeated trials only, there was a significant main effect from baseline [*F*_(1,6.542)_ = 7.0399, *p* = 0.035] and an interaction between treatment and day [*F*(_16,80_) = 3.468, *p* = 0.0001]. For non-repeated trials, there were no significant main effects or interactions.

**FIGURE 4:**
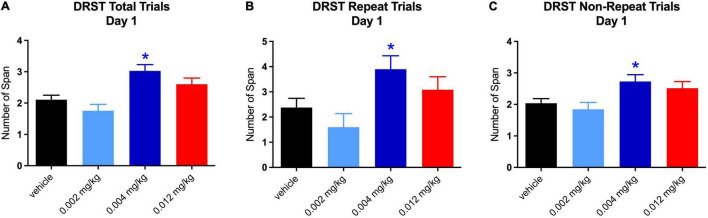
Effect of PF-6294 on DRST Spatial Performance on Day 1. **(A–C)** The increase in total span on day 1 at the middle dose was driven by increases on both repeated (*p = 0.040) and non-repeated trials (*p = 0.019). Error bars = SEM.

**FIGURE 5:**
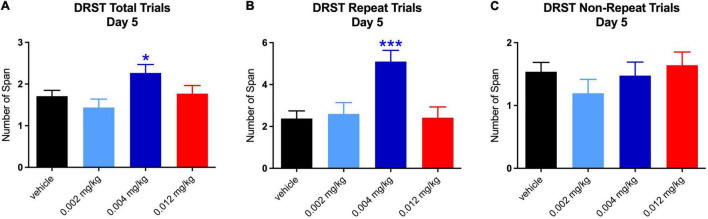
Effect of PF-6294 on DRST Spatial Performance on Day 5. **(A–C)** The increase in total span on day 5 for the middle dose (*p = 0.035) was seen only on repeated trials (***p = 0.0001). Error bars = SEM.

The increase in total span on day 1 at the middle dose was driven by increases on both repeated [*t*(_119_._96_) = 2.482, *p* = 0.040] and non-repeated trials [*t*(_120_) = 2.759, *p* = 0.019; see Figures 4A–C]. It is not surprising that there was an equal effect of the repeated and non-repeated trials at Day 1 since this is the 1st time the repeated trial was administered and therefore was not qualitatively different from the non-repeat trials. However, the increase in total span on day 5 for the middle dose was seen only on repeated trials [*t*(_119.26_) = 4.442, *p* = 0.0001; see Figures 5A–C]. Despite the highest dose producing an increase in total span on day 4, when tested separately, neither repeated nor non-repeated trials on that day were significantly different from vehicle.

## Discussion

In the current study, we demonstrated that there was a significant effect of a novel selective, potent, non-catechol D1R agonist (PF-6294, Pfizer, Inc.) on a task of spatial working memory (DRST). Specifically, the middle range dose resulted in a significantly higher total memory span when compared to vehicle. These findings are of particular interest given that the DRST relies on the PFC for normal performance (unpublished data – dorsolateral prefrontal cortex lesions in young monkeys significantly impairs performance on DRST) and this is a cortical area that shows age-related changes in DA levels. Further, PF-6294 showed a non-linear dose response on the DRST, a pattern which has been reported previously in studies examining the impact of D1 agonist pharmacology on preclinical cognitive tasks (Arnsten et al., 1994, 2015; Cai and Arnsten, 1997; Wang et al., 2019).

In contrast, there was no effect on performance on the DNMS task (basic acquisition or delay condition). However, unlike the DRST, the DNMS task, in particular the re-acquisition and delay phases of the task, are considered to rely more on the medial temporal lobe (Rapp and Amaral, 1989, 1991; Beason-Held et al., 1999). Further, as the DRST task was the 3rd task administered in each testing session, the unique effect on the performance of this task may be the result of the cumulative dosing that occurred prior to administration of the DRST task. Future studies with counterbalancing of task administration (eg. administration of DRST at week 1 and DNMS at week 3) may provide additional insight into the effect of DA agonists on DNMS performance.

### Cognition and the Prefrontal Cortex

It is well established that normal aging is characterized by declines in short-term memory, psychomotoric speed, naming, and executive function (Albert, 1993; Bachevalier, 1993; Arnsten et al., 1994; Arnsten and Jentsch, 1997; Herndon et al., 1997; Cotman et al., 2002; Moore et al., 2003, 2005, 2006; Fisk and Sharp, 2004; Drag and Bieliauskas, 2010; Alexander et al., 2012; Darusman et al., 2014) in both humans and monkeys. Of these cognitive abilities, executive function (EF) is one of earliest cognitive domains to evidence change in humans (Fisk and Sharp, 2004; Finch, 2009; Drag and Bieliauskas, 2010) and non-human primates (Bachevalier, 1993; Lai et al., 1995; Herndon et al., 1997; Moore et al., 2003, 2006; Darusman et al., 2014). Spatial working memory, a component of EF, is mediated in part by the prefrontal cortex (PFC) (Raz et al., 1997; Moore et al., 2009; McEwen and Morrison, 2013; Borella et al., 2014; Toepper et al., 2014; Arshad et al., 2016; Johnson et al., 2016; Kwon et al., 2016; Funahashi, 2017) which is one of the first cortical regions to show age-related change. For example, magnetic resonance imaging studies have demonstrated that age-related atrophy is particularly prominent in the frontal lobes (Raz et al., 1997; Salat et al., 1999; Tisserand et al., 2002; Gunning-Dixon and Raz, 2003). Further, data from our laboratory and others over the past several decades has demonstrated an increase in neuroglial cells with dark cytoplasm and an increase in the amount of inclusion material in the neuroglial cells in the PFC, and a decrease in the thickness of layer 1 in area 46 of aged monkeys (Peters et al., 1994; Guttmann et al., 1998; Peters and Sethares, 2002; Makris et al., 2007; Wisco et al., 2008; Drag and Bieliauskas, 2010). In addition, degenerative changes in the myelin and underlying white matter occurs in the PFC of aged monkeys and correlates with cognitive performance (Peters et al., 1994). In addition, there is an increase in phagocytic and activated microglial in frontal white matter (Shobin et al., 2017), a loss of myelin integrity in area 46 as measured by decreased fractional anisotropy in PFC white matter (Makris et al., 2007), and an overall decrease in the volume of white matter with age (Wisco et al., 2008). Taken together, these findings show a particular vulnerability of the PFC to age-related alterations in morphology that are related to declines in cognitive function.

### Dopamine, Prefrontal Cortex, and Aging

The PFC is a main target of the mesocortical DA system and within this region dopamine is present in a higher concentration than in any other cortical region (Brown et al., 1975; Bannon and Roth, 1983; Felten and Sladek, 1983; Berridge et al., 1993). The PFC DA system is unique in being characterized by a high DA turnover rate, higher and more irregular neuronal firing, and a lack of autoreceptors (Bannon and Roth, 1983; Tam et al., 1990). Dopamine afferents in the human and non-human primate PFC have a bilaminar distribution, with the highest concentrations in layers I, II, III, and V, VI of the cortex. In terms of DA receptors, DA D1 receptors are approximately tenfold more concentrated in the PFC than the D2 receptors and are located primarily in the superficial layers of cortex (layer I, II, and IIIa) (Savasta et al., 1986; Goldman-Rakic et al., 1990, 1992; Lidow et al., 1991; Sawaguchi and Goldman-Rakic, 1991; Smiley et al., 1994).

Neurochemical studies have demonstrated that dopamine (DA) plays a role in the cognitive functions subserved by the PFC (Brozoski et al., 1979; Sawaguchi and Goldman-Rakic, 1991; Goldman-Rakic et al., 1992; Arnsten et al., 1994; Castner and Goldman-Rakic, 2004). However, levels of DA are reduced in both aged monkeys and humans, with the most significant reductions occurring in the prefrontal and temporal cortices (Goldman-Rakic and Brown, 1981; Goldman-Rakic et al., 1990; Arnsten et al., 1994, 2015; Castner and Goldman-Rakic, 2004; Wang et al., 2019). In aged monkeys, DA levels in the PFC decrease by 56% (Goldman-Rakic and Brown, 1981; Arnsten et al., 1994) and there is evidence of a decrease in DA receptors in the brain regions including the PFC (Rinne et al., 1990, 1993; Suhara et al., 1991; Arnsten, 1993; Inoue et al., 2001; O’Brien et al., 2003). A study from our laboratory demonstrated no age-related changes in post-synaptic D1 receptor binding in the PFC, which is in contrast to studies in the literature (Suhara et al., 1991; Arnsten, 1993). However, the sample size in our former study was small (8 young and 8 aged) and there was significant variability in binding density in the aged monkeys, both of which may have contributed to the lack of significant differences between age groups. Of particular interest though, we showed that there was a significant correlation between D1 receptor binding density and age-related impairment on the DRST task, with aged monkeys having the lowest spans on the DRST and D1 receptor binding density in dorsolateral PFC regions (Moore et al., 2005). This is particularily relevant, as the DRST task is the same task used in the present study on which aged monkeys demonstrated improved performance while receiving PF-6294. These promising findings build on similar studies in the field (Yohn et al., 2015; Wang et al., 2019) and provide further evidence of the efficacy of D1R agonists to effect the cognitive processes of the PFC in aging and perhaps disease processes that involve the PFC.

Further, it would be of interest in future studies to include young monkeys to determine the effect of non-catechol D1R agonists in younger monkeys as there are variable results in the literature. First, a recent study assessed the impact of a non-catechol D1R agonist similar to PF-6294 and demonstrated that young monkeys appeared to benefit from a low dose and aged monkeys benefited from a high dose (Williams et al., 2017). However, Arnsten et al. (1994) demonstrated that administration of the D1 antagonist, SCH-23390, to young monkeys caused impairments on a delayed response task but did not impair performance of aged monkeys. Therefore, future studies with PF-6294 should include young monkeys in order to determine its effect on a young brain not naturally depleted of DA.

### Dopamine Receptor Agonists and Aging

Several studies have provided extensive evidence of a link between age related changes in dopamine receptor activity in various cortical regions, including the PFC, and performance on neuropsychological tests (Volkow et al., 2000; Kaasinen and Rinne, 2002; Wang et al., 2019) Arnsten et al. (1994) demonstrated that both a partial DA D1 agonist (SKF38393) and a full DA D1 agonist (dihydrexidine) improved performance by aged monkeys on the Delayed Response Task (DR - a working memory task). Further, the improved performance was reduced by the administration of the DA D1 antagonist, SCH23390. Similarly, Cai and Arnsten (1997), showed that the DA D1 full agonists, at low doses improved performance on the DR task which also was reversed by the DA D1 antagonist, SCH23390.

Yohn et al. (2015) demonstrated that the administration of DA D1 agonists, SKF38393, SKF81297 and A77636 reversed the negative effects of the DA D1 antagonist in rats while performing the T-Maze task. These findings reinforce the notion that the DA D1 receptors are functionally critical for successful completion of cognitive tasks.

Despite these extensive findings, a long-standing issue with the use of DA D1 agonists is that the selective pharmacological tools available are catechol-based molecules which are rapidly metabolized and desensitize the receptor quickly and therefore have a reduced effect over time. Recently, Gray et al. (2018), demonstrated a new class of selective, potent non-catechol DA D1 receptors agonists and partial agonists which are reported to have favorable *in vivo* pharmacokinetics, and reduced receptor desensitization. The D1-mediated pharmacology of compounds from this class were demonstrated in Wang et al. (2019) who showed that administration of the novel non-catechol D1R agonist, PF-3628, had an excitatory effect on working memory circuits in the PFC when directly applied via iontophoresis to aged rhesus monkeys. The administration of PF-3628 produced an inverted-U dose response curve, similar to the response observed in the current study, with increased firing of dorsolateral PFC neurons at low to moderated dose, especially in the oldest monkeys in the study. Of particular interest was the dorsolateral PFC neurons excited by PF-328 are in a cortical region involved in working memory. In the current study, PF-6294 likely resulted in a similar restoration of excitatory action of DA neurons in the PFC and that could explain the improved performance on the DRST task. The dose response observed in these two studies adds to a body of evidence that cognitive benefits of D1 agonism may require an optimal level of dopamine system activation in order to enhance cognitive function and should continue to be explored.

## Conclusion

The current study has demonstrated that the administration of the non-catechol DA D1 agonist, PF-6294, enhances performance on the DRST, a spatial working memory task, in aged rhesus monkeys. Though the significant effect was only observed on the 1st and last days of testing, these results are consistent with other recent studies demonstrating the efficacy of this class of non-catechol DA D1 agonists to have sustained efficacy (Wang et al., 2019). While this study utilized a small number of monkeys, the positive effect of the administration of the novel selective, potent, non-catechol DA D1 agonist PF-6294 adds to a growing body of literature supporting the therapeutic value of this class of agonists to slow or reverse age-related cognitive decline and warrants further study.

## Data Availability Statement

The raw data supporting the conclusions of this article will be made available by the authors, without undue reservation.

## Ethics Statement

The animal study was reviewed and approved by IACUC Boston University.

## Author Contributions

TM, RK, and RJK contributed to conception and design of the study. DY performed the statistical analysis. TM, RK, RJK, and DY wrote the manuscript. All authors contributed to manuscript review and revision and read and approved the submitted version.

## Conflict of Interest

DY, KF, DV, DG, RB-G, and RK were employed by Pfizer, Inc., United States. The remaining authors declare that the research was conducted in the absence of any commercial or financial relationships that could be construed as a potential conflict of interest.

## Publisher’s Note

All claims expressed in this article are solely those of the authors and do not necessarily represent those of their affiliated organizations, or those of the publisher, the editors and the reviewers. Any product that may be evaluated in this article, or claim that may be made by its manufacturer, is not guaranteed or endorsed by the publisher.
